# Psychologists’ experience of a malpractice complaint: Their relationship with and processes at the regulator

**DOI:** 10.4102/hsag.v25i0.1384

**Published:** 2020-10-12

**Authors:** Hanlé Kirkcaldy, Esmé van Rensburg, Kobus du Plooy

**Affiliations:** 1 School of Psychosocial and Behavioural Sciences, North-West University, Potchefstroom, South Africa

**Keywords:** disciplinary action, ethics, malpractice complaints, psychologists, registration body, regulator

## Abstract

**Background:**

Professional malpractice complaints in the South African health arena have increased over the last decade. There is a lack of research on how South African health practitioners experience professional malpractice complaints and complaint processes.

**Aim:**

This article reports on one aspect of the findings in a more extensive study relating to the complaint experience of psychology practitioners, namely how a group of psychology practitioners experienced their relationship with and the processes at the regulator during a malpractice complaint. The regulator refers to the professional registration body which manages complaints against practitioners.

**Setting:**

The study included 10 registered South African psychologists who experienced a malpractice complaint.

**Methods:**

After sampling, semi-structured interviews were conducted, audio-recorded and transcribed. The data were managed using interpretative phenomenological analysis (IPA) to elicit the personal, subjective experience of the individual participants.

**Findings:**

Two superordinate themes and related subthemes emerged from the analysis. First, relating to the experience of the complaint procedures and processes, participants experienced an extended timeframe for complaint management, a lack of communication during complaint management, legal challenges during some disciplinary proceedings and some complaints as unjustified and frivolous. Second, participants were unsure of their relationship with the regulator. Their responses denoted instances of vulnerability and inequality during proceedings.

**Conclusions:**

The findings call for closer collaboration between the registration body and practitioners during complaints management, to eliminate vexatious complaints, to streamline processes and to encourage guidance of and support for the professional.

## Introduction

Reports on professional healthcare issues indicate that South Africa has experienced a sharp escalation in medical malpractice litigation over the last decade (Howarth & Hallinan 2016; Malherbe 2013). This burdens already strained state resources to manage large compensatory expenditures and implies increased medical malpractice insurance fees for practitioners in the private sector (Malherbe 2013; Pepper & Nöthling Slabbert 2011). Patients should undoubtedly have the right to complain and be compensated in the case of clinical negligence. Still at the same time, a robust and reliable system of defence and protection should also be available to healthcare practitioners (Howarth & Hallinan 2016).

### The role of a professional regulator to protect the public

Complaints against registered healthcare practitioners are usually managed by a professional regulator. The Health Professions Council of South Africa (HPCSA) is the regulator for all registered healthcare professionals and as a statutory body governs the professions^1^ in terms of the *Health Professions Act*, Act 56 of 1974 (*Health Professions Act, 56/1974*). Regulation of the professions by a professional body that operates with the authority of the state is essential to protect the public against negligent, unscrupulous, unethical or impaired professionals, to uphold the public reputation of the profession and protect against those who do not meet the required registration standards to belong to the profession (Allan 2016).

Succinctly stated, the complaints process can be described as follows: When a member of the public complains about the professional conduct of a healthcare practitioner, the registrar forwards the complaint to the relevant professional body for investigation. A preliminary committee of inquiry, thereafter, requests practitioners and their legal representatives to respond to the complaint. If the explanation offered by the practitioner is not satisfactory, a professional conduct inquiry could be convened (*Health Professions Act*, 56/1974, HPCSA 2019c).

A delicate balancing act exists for the regulator in terms of ‘protecting the public and guiding the professions’ (HPCSA 2019a). It is possible that the role – if not verdict – of the regulator when investigating a complaint and perhaps having to sanction a professional may lean towards concern for and protection of the public. This role and the consequences thereof may have unforeseen detrimental effects on the clinician.

### The effects of a complaint on healthcare professionals

Research indicates that the mental and physical health of practitioners remains at risk during a malpractice complaint. Regardless of their profession, practitioners experience a professional malpractice complaint as emotionally and physically challenging. Medical specialists, general practitioners, physiotherapists and psychologists reported feelings of depression, dysphoria, insecurity and fear during a complaint (Verhoef et al. 2015). Medical practitioners in the United Kingdom who endured a complaint were 77% more likely to suffer from moderate to severe depression, had double the risk of developing anxiety when compared to their colleagues who had never endured a complaint and were 2.08 times more likely to have suicidal ideation or thoughts of self-harm (Bourne et al. 2015).

Certain practitioners suffered a type of post-traumatic stress disorder known as medical malpractice stress syndrome (MMSS) (Paterick et al. 2017). Medical malpractice stress syndrome, sometimes also referred to as clinical judicial stress syndrome (Arimany-Manso, Vizcaíno & Gómez-Durán 2018), is a set of physical, mental and behavioural symptoms as manifestation of the acute and chronic stress and psychological trauma experienced by practitioners who faced the unique difficulties of malpractice litigation. After receiving a complaint, practitioners were apprehensive about the advent of additional complaints and reacted with defensive practices; they felt exposed and unsafe if the process was reported in the media, and if the disciplinary findings were published along with their personal details (Verhoef et al. 2015). Moving to the field of psychology in South Africa, Kirkcaldy, Van Rensburg and Du Plooy (submitted for review) investigated the experience of psychologists accused of professional malpractice and found adverse psychological and physical effects in the wake of the complaint. The practitioners mainly reported experiencing shock, anxiety, fearing the loss of their livelihoods, fearful anticipation of future complaints (with a sense of dread and worry), experiencing various physical symptoms necessitating medical attention, growing isolation, self-doubt and fearful concern over perceived professional reputations and integrity (Kirkcaldy et al. submitted for review).

### The relationship between professionals and the regulator

The relationship between the professional and regulator may be fragile and difficult during a complaint process, perhaps as the professional receiving the complaint expects support or guidance from the regulatory body while already combatting the emotional shock of receiving a complaint. The role of the regulator to guide, support and advise the professional may, however, not be central during a disciplinary hearing. Notwithstanding, the ideal is that ethics committees and regulators do not take a punitive stance in disciplinary matters, but rather ‘encourage wrongdoers to learn from their mistakes’ (Allan 2016:110). It is hypothesised that if the professional does not experience a sense of support, guidance and learning during this process, this may lead to an intensified detrimental impact on the mental and/or physical health of the practitioner.

Furthermore, Van der Merwe (2010) found that psychologists had an implicit expectation and need for more prominent and practical guidance by the professional regulator and also experienced frustration with various general processes and procedures. The HPCSA has recently embarked on a ‘turnaround project’ to improve its processes and procedures and to collaborate more closely with the professions (HPCSA 2018; South African Government News Agency 2019). One of the aims stated in the recent HPCSA annual report is to ‘restore the integrity of the system for professional conduct inquiries’ (HPCSA 2018:10). This step is welcomed and indicative of a move by the regulator to improve these processes.

The findings reported here aim to contribute to a possible transformational discourse between practitioners and the HPCSA. In light of the sensitive relationship between the professional regulator and the professional during disciplinary action and given that both the complainant and practitioner have rights in the matter as encapsulated in the South African Bill of Rights (The Bill of Rights 1996), we approach this issue mindful of balancing the ethical principles of non-maleficence and justice.

## Research methods and study design

The findings reported here ensued from research in a comprehensive study focussing on the personal experience, coping and meaning-making of psychologists after a malpractice complaint (Kirkcaldy 2020). The larger study set out to explore and elicit the claims and concerns of practitioners subjected to a malpractice process and to make sense of their experience by interpreting their individual accounts, exploring their coping strategies and the meanings made from this experience. We report on the coping strategies elsewhere (Kirkcaldy, Van Rensburg & Du Plooy 2020). However, it was found that practitioners not only reported on their personal emotional and physical experiences, how they coped and the subsequent growth they experienced, but also used the interviews as an opportunity to voice their experience of and relationship to the regulatory body during complaint management (Kirkcaldy, 2020). This provided the opportunity to examine the unique relationship between the regulator and the professionals during a disciplinary process in greater detail.

As a qualitative research approach, the epistemological stance in interpretative phenomenological analysis (IPA) remains interpretative, hermeneutic and phenomenological (Larkin & Thompson 2012). ‘How’ people relate to the world and to the events they experience, and the meanings they make of the experience, is elicited from their particular and detailed idiographic accounts. These experiences are thereafter linked to psychological concepts veiled in their narrative (Larkin & Thompson 2012). This is known as the double hermeneutic: the researcher endeavours to make sense of the participants making sense of their experiences (Smith, Flowers & Larkin 2009). As such, it is mostly an inductive process of analysis, whereby the participants and the researcher develop patterns of meaning from a particular set of cases (Smith et al. 2009).

### Setting

The research setting and study population comprised of registered South African psychologists who were subjected to a professional malpractice complaint during their careers. To participate in the study, practitioners must have experienced a completed complaint process at least once in their careers.

### Study population and sampling strategy

A general invitational email was sent to a large group of psychologists whose email addresses appeared in the public domain. Practitioners who volunteered by responding to this email received an information package. Interested parties proceeded to the informed consent process.

This purposive sampling strategy (Strydom & Delport 2011) was relied upon, as only psychologists having undergone a complaint process would provide typical, representative attributes of the phenomenon under scrutiny. Snowball sampling, where participants already recruited for the study could indicate other possible participants, was allowed (Strydom & Delport 2011). Ten participants (female = 8) were included in the final study, with an average age of 57.7 years (SD = 8.09) and an average of 23.2 years of experience (SD = 8.68). Participants were drawn from the clinical, counselling and educational registration categories in psychology. Some had endured several complaints, whereas others were subjected to only one complaint. The categories of complaints ranged from work in the medico- and psycho-legal and therapeutic areas of practice. The timespan of complaints ranged from more than 10 years ago to as recently as a few months before the interviews. Some practitioners who received multiple complaints, specifically in the area of medico- and psycho-legal work, were managing ongoing complaints, but these cases did not form part of the interviews and discussions.

Qualitative studies typically involve small sample sizes because of the individual, detailed and idiographic approach, and a sample size of three to six participants or conducting 4 to 10 interviews is described to be adequate in an IPA study (Smith et al. 2009).

### Data collection

Data collection occurred by way of audio-recorded, semi-structured, in-depth interviews during the period of July 2017 to December 2018. Smith et al. (2009) endorse the use of semi-structured interviews in IPA studies to collect detailed and in-depth data while setting a ‘loose agenda’ in order to achieve the purpose of the interview (p. 58). Probing questions and clarification of statements were allowed, as this demonstrates appropriate responsiveness to participant accounts and an openness to follow emerging topics that seem important to the participants (Nieuwenhuis 2016).

Recruitment and interviews continued until data saturation was reached. The latter occurred when no significantly new ideas or new data emerged from the continued analysis of the interview transcripts, and the theoretical concepts seemed to have been adequately developed (Nieuwenhuis 2016). Nine interviews were conducted at the home or office of the participants, and one interview was conducted at the office of the first author.

### Data analysis

Interpretative phenomenological analysis, a qualitative research approach that has a phenomenological focus and an interpretative stance, was used to analyse the data (Larkin & Thompson 2012; Smith et al. 2009). Continued supervision and collaboration between the authors were used to improve the plausibility of the analysis and reliability of the results (Larkin & Thompson 2012).

### Trustworthiness

It is important to prove trustworthiness and rigour in qualitative studies. We utilised strategies such as those recommended by Forero et al. (2018). Detailed track records of the raw data transcripts, data collection processes and working drafts of the study protocols were kept throughout. This created a record of the decisions made while doing the analysis and created an audit trail, which informed dependability (Forero et al. 2018). Credibility was enhanced by the subsequent succinct, authentic and in-depth accounts obtained during the lengthy interviews from the homogenous, purposive sample of experienced participants who had the opportunity to review the transcribed interviews and make any changes and additions. Frequent cross-checking of findings between the authors and a commitment to a close understanding of the particular and subjective experience as expressed by the participants, improved the validity of the findings (Forero et al. 2018).

### Ethical considerations

The study was approved by the Health Research Ethics Committee (HREC) of the North-West University, approval number NWU-00367-16-S1. The informed consent process and documentation were managed by a registered psychologist who acted as a mediator and who signed a confidentiality agreement. The use of a mediator ensured that participants were not coerced into participation after the initial contact with the researcher and guaranteed that they could withdraw from the study at any time without obligations. The interviews were manually transcribed by the first author. To protect the identity of the participants, protocols were rendered anonymous immediately by assigning a pseudonym to each participant during the interview and subsequently a number to each transcribed protocol. A free consultation with a registered psychologist was offered to all participants in the event of any distress elicited as a result of the interview.

## Results

The findings are reported using verbatim quotations or part-quotations by the participants. As such, idiosyncratic language use and small grammatical errors are retained in the quotations to honour the authentic reports from the participants. Table 1 summarises the findings.

**TABLE 1:** Themes and subthemes relating to the findings of the relationship with and experiences of the regulator during a professional malpractice complaint process.

Themes
**Theme 1: The experience of the procedures and complaint processes at the registration body**
The participants experienced an extended timeframe for complaint management
The participants experienced a lack of communication during complaint management
The participants experienced legal challenges during some disciplinary procedures
The participants experienced some complaints as unjustified and frivolous
**Theme 2: The par ticipants experienced uncertainty in their relationship with the regulator**
The participants experienced vulnerability
The participants experienced a sense of harm and inequality

### Theme 1: The experience of the procedures and complaint processes at the registration body

Four subthemes emerged relating to the experience of the procedures and the complaints processes, namely the experience of an extended timeframe, a lack of communication, some legal challenges and the perception of unjustified complaints. These are discussed below in more detail.

#### The participants experienced an extended timeframe for complaint management

Every participant noted the extended period in which complaints were managed and concluded. P3 (female, 55) reported, ‘It was actually about fourteen months before it was sorted out’. Others described an even longer period, such as P8 (female, 58) who described a case ‘going on for four, five years’ and P7 (male, 45) who declared that the duration of his case ‘was about four years, from start to finish’.

The meaning of extended time and frequent postponements was that the participants experienced these as exacerbating the problematic nature of the complaint experience, leading to uncertainty and vulnerability. P6 (male, 70) said: ‘In that time you don’t know what’s going to happen to you professionally’. P2 (female, 62) expressed that inexplicable and sudden postponements of hearing dates ‘felt abusive’. P1 (female, 54) felt that the extended period affected not only the practitioner, but also the complainant: ‘It’s terrible for the people who laid the complaint too, because in their minds maybe they do think they have a justified complaint’. This demonstrated empathy to the complainant and a mature understanding of the impact of the extended process on all parties.

#### The participants experienced a lack of communication during complaint management

Eight of the participants expressed a need for swift, clear and continuous communication from the regulator during the complaints process and were disappointed when this did not occur. P1 described that she asked for advice from the regulator after receiving a complaint: ‘I’ve phoned them, e-mailed them, and said this is the scenario, what do you recommend?’ She reports receiving the response that to comment on her questions ‘will take too long to put it in an e-mail’. P10 (female, 59) similarly remembered: ‘At the time I wrote … and asked them a few questions, but I never got a response’. The experience of not being able to access an officer at the regulator who could offer procedural guidance or advice regarding the complaint and its related processes was interpreted by the practitioners as invalidating or indifferent during an already vulnerable and anxiety provoking time.

#### The participants experienced legal challenges during some disciplinary procedures

Participants described modern-day practice as having an intense legal atmosphere and that this environment contributes to the possibility of complaints. In particular, participants who engaged in psycho-legal/medico-legal work experienced themselves as the subjects of legal strategising and attempts to discredit their work as part of the cases in which they were involved. P1 revealed:

‘As soon as you release a report, one of the parties is going to report you to the HPCSA, because they want to be able to say in court, but she’s been reported to the HPCSA.’ (female, 54)

P7 concurred and said: ‘Prior to my evidence they then started to say, basically threatened, that they are going to report me if I go ahead and testify’.

Contrary to these calculated legal strategies when bringing complaints against practitioners, participants in this study expressed concern about the levels of legal awareness and circumspection during some disciplinary procedures. P7 felt that procedural errors during hearings had to be pointed out by the legal experts representing the various parties. He related that ‘on the first day the charge sheet was wrong’ that led to an inconvenient postponement and on another occasion ‘a biased statement … was made during the adjournment’ that was taken up by a legal representative. He further remarked that in his experience ‘court cases at least have very strict laws about how they operate … the disciplinary hearings are far more fluid and [*do*] not [*have*] hard and fast rules’. This induced feelings of uncertainty and vulnerability during the proceedings.

Participants 1, 2, 4, 6, 7 and 8 felt that if the complaint is related to the medico- or psycho-legal field, members of the disciplinary committee and experts called to testify in those cases should have specific expertise in these arenas. P8 believed ‘you should be judged … by your peers’. Relating to expert witnesses, P2 felt alarmed about the expert called to advise the committee on her case: ‘I read her report … she doesn’t understand … she doesn’t do this work properly’.

#### The participants experienced some complaints as unjustified and frivolous

Complaint documents are usually complex and constructed of many elements. Participants experienced frustration at having to respond to what they felt were at times ungrounded complaints or untrue statements within the complaints. P5 (female, 45) judged that ‘it was completely unfair that this [*the complaint*] had gone to the Health Professions Council’ because of a challenging complainant, while P7 was ‘confident that what I’d done was right, but still if you have a board that doesn’t understand it, you can be right, but they can find you disciplinarily wrong’. This was also endorsed by P4 (female, 56) who said: ‘On the one hand, it was all so unnecessary, and there was this injustice to it … because you know you’re innocent’. Most of the participants experienced frustration that some complaints, which in their view were vexatious or even false, could still proceed over an extended period.

Responding to baseless complaints meant that the practitioners were still subjected to the personal, physical and economical adversarial effects of their involvement in these complaints. P1 experienced this as instances where ‘the system is abused’ by complainants. P6 explained that enduring a complaint that had no merit meant ‘I’ve still got to answer that complaint and I’m still facing the same questions as a person who sleeps with his patients’. Participants expressed a strong need for an initial process that could screen for and eliminate unnecessary, possibly vexatious, time-consuming and potentially damaging complaints if these had no grounds to proceed. The meaning of these experiences could be that practitioners felt perplexed by the vagueness surrounding the interpretation of misconduct by their regulator and exposed to the manipulations of certain clients – stuck in the middle with nowhere to turn.

### Theme 2: The participants experienced uncertainty in their relationship with the regulator

The second theme elicited from the data, related to the practitioners experiencing a sense of uncertainty in their relationship with the regulator and could be divided into two sub-subthemes: first, experiencing a sense of vulnerability and second, experiencing a sense of harm and inequality.

#### The participants experienced vulnerability

Referring to the perception that false and vexatious complaints could proceed, the participants felt unprotected from and vulnerable to the regulator. P7 appealed to the registration body and said: ‘[*you’re*] supposed to protect us … if you guys don’t, then by God, who does?’ P6 articulated: ‘I think that we need something that protects us, to feel that there’s somebody out there, really, really looking to understand … the legitimacy of the compliant’. P4 explained: ‘You don’t feel that the body that’s supposed to protect you is protecting you, and that is a sell-out’. Most of the participants had the perception that the public received the protection during a complaint process, whereas the professional was an outsider to this process. Participants 1, 3, 4, 6 and 10 mentioned a need for guidance and support for the professional in this situation.

Participants also experienced vulnerability and disempowerment during the process. P9 described:

‘[*T*]he powerlessness, the terrible powerlessness … you cannot prove something [to the disciplinary committee] if they were not there. It’s just me and my word and my word wasn’t good enough. … I left my self-confidence in that room.’ (female, 70)

P8 said, ‘I should actually mention the fear we have of the Board of Psychology … it’s just such a bad situation’. This may mean that practitioners had expectations of protection and security during the complaint process, which were not met.

#### The participants experienced a sense of harm and inequality

Participants generally reflected on issues of guilt or innocence and reported experiencing a state of accusation, rather than investigation. P3 said: ‘We are treated as criminals … they assume you’re guilty’ [*before evaluating the merits of the complaint*]. She experienced the eventual guilty verdict against her as having a ‘criminal record’ which will follow her around for the rest of her career. P9 said that she felt in a ‘state of accusation’ and like ‘the offender’. P2 declared that she felt like the accused facing her ‘accuser’. This may mean that regardless of the verdict, there was perceived harm to professional reputations simply by receiving a complaint.

It was, therefore, important for participants to have their name cleared and to have public records corrected. P6 related how he felt a sense of vindication only when the complainant was reprimanded by the presiding officer in one of the cases, while P2 declared, ‘I didn’t feel relief until there was that stamp of approval’ [*referring to being found not guilty*].

Contrary to these experiences, P2 and P8 both had a hearing where they felt an absence of this perceived inequality. P2 described a preliminary hearing where ‘… I sat with them, I found [*it was*] fine, it was convivial, it was a conversation, it was a discussion … it was collegial’. Both strongly preferred this egalitarian approach where they felt part of the investigation into the complaint.

## Discussion

The experience of the processes at and the relationship with the registration body in a group of psychologists during a professional misconduct charge is reported in this study.

### Key findings

The findings indicate that the participants in this study experienced administrative frustrations and legal challenges during the complaint process. Furthermore, they experienced apprehension and trepidation about facing what was perceived as vexatious or frivolous complaints and anxiety about their relationship with the regulator.

### Discussion of key findings

Professional negligence is ‘a statistically inevitable event’ (Allan 2016:105) and could happen to the most conscientious, diligent and well-meaning practitioner. The likelihood of a complaint lodged against any healthcare practitioner including psychologists in the South African context, is therefore highly probable. The management of unsubstantiated complaints was of great concern to the participants in this study, particularly in cases related to medico- and psycho-legal work. It therefore seems practical to refine the existing systems at the regulator to identify and eliminate vexatious complaints. More extensive consultation than is currently the case may be called for with experts in the field and with the implicated practitioner. Any professional malpractice complaint has the potential to cause considerable harm to the professional reputation and identity of a practitioner and, depending on the nature of the cases, some complaints may have more to do with strategic moves and countermoves by litigators or with compensation possibilities, than true competence or incompetence (Charles 2001; Woody 2009).

Unsubstantiated complaints in psychology could sometimes be made as an attempt by service users to blame others or to seek a sense of retribution for outcomes that were not in their favour – perhaps more so in the event of medico- or psycho-legal assessments (Thompson 2007). Complaints to the regulator may also be used to test the potential for success in planned legal procedures of a civil nature (Oosthuizen & Carstens 2015). In contrast, practitioners are more concerned about their reputation than about the financial implications of a complaint. Therefore, every unsubstantiated complaint becomes a massive source of distress (Burkle, Martin & Keegan 2012; Thompson 2007).

Most of the participants in this study experienced the complaint process as extended, slow as well as fiscally and temporally expensive. They experienced a need for ‘strategizing’ complaints to be filtered out before they reach the practitioner and mandated a response. In establishing the prima facie merits of a case, the participants in this study pleaded for a changed approach, namely a collaborative and egalitarian process in conjunction with the committee of the preliminary inquiry, or the more robust intervention of a mediator or an officer at the regulator, such as the ombudsman. International and local experts have recommended methods to minimise frivolous complaints: due-process protection for practitioners working in high-risk areas such as child custody and medico- or psycho-legal evaluations; and alternative claim resolution strategies such as arbitration and mediation (Benesch 2011; Claassen 2016; Crane 2012; Schoenfeld, Hatch & Gonzales 2001; Williams 2001).

The theme of explicating the relationship between the healthcare professional and the regulator is a sensitive topic. It is, however, not only of local interest but has received international attention (Professional Standards Authority (PSA) 2015, 2016; Schoenfeld et al. 2001). On the one hand, during a disciplinary hearing, it is understandable that the protection of the public and the regulation of professional standards are the primary mandate of a regulator rather than the beneficial guidance of the implicated professional. This role is important and indispensable. On the other hand, the expectations of the participants in this study as representatives of the psychology profession were that preliminary inquiries and disciplinary hearings (if the complaint warrants an inquiry) should be more expedient, standardised, legally reliable, psychologically beneficial and educationally constructive. These concerns should receive serious consideration by the regulator as part of its new ‘turnaround project’ as referred to earlier that aims to improve its processes and procedures (HPCSA 2018; South African Government News Agency 2019).

Recommendations for the creation of a more supportive and less punitive complaint process are endorsed by authorities and researchers alike (PSA 2016; Schoenfeld et al. 2001). Furthermore, engaging professionals in training about preventative actions, the promotion of best practices or ‘doing what works’ could further reduce harm to patients and be more cost-effective than protracted disciplinary hearings (PSA 2016).

Professional registration bodies could be regarded as extensions of the state as they are regulatory authorities and administrative tribunals that exercise power through legislation (Redelinghuys, Bütow & Carstens 2006). To expect members of disciplinary committees to be both protectors of the professional while at the same time disciplining them may amount to *a dual role*. In the United Kingdom, reports suggested that the sanctioning role of the regulator, evaluations regarding fitness to practice, the maintenance of professional standards, the inspection of education standards and the prevention of harm will always be the primary focus of the regulator (PSA 2016). However, the expedient resolution of complaints was also encouraged in the reports, as well as managing complaints on an operational level by the professionals themselves, their employers or through mediation where possible (PSA 2016). Howarth et al. (2015) made similar recommendations for healthcare professionals in South Africa recommending a ‘good complaints system’ in practices and organisations to assist clients to air their concerns and dissatisfactions and to manage complaints before these escalate to the regulator or a civil court (Howarth et al. 2015).

### Strengths and limitations

The limitation of most qualitative studies lies in the small sample sizes and the necessity of purposive sampling, which creates transferability problems. However, empirical transferability to other settings was not the aim. The strength of this study lies therein that it is firmly grounded in a South African sample, and the findings therefore have direct implications for the South African regulator and South African practitioners. It will be possible for practitioners to compare their own experience with those of the participants in this study and reflect on the meaning and implications of the outcomes.

### Recommendations

As found elsewhere, the South African professional landscape may be in need of more active and robust peer support networks (PSA 2015) where best practices and the management of potential complex cases that could lead to complaints can be reflected upon and discussed. Internationally, there have been several successful examples of professional advocacy networks (Peterson 2001), standing committees on board disputes (Williams 2001) or collegial provider networks (Couch & Thiebaud 2002) that support practitioners, and these are activated the moment a complaint is lodged against a healthcare professional. Psychological and professional services are helpful during and after the complaint and should also be routinely recommended to practitioners receiving a complaint (Schoenfeld et al. 2001). This form of support could possibly protect against the development of MMSS.

The participants in this study recommended a remodelling of particularly the initial phases of a complaint process at the regulator, and the institution of processes that can eliminate unsubstantiated complaints. Ideally, this should be done as early as possible especially in cases involving medico- and psycho-legal work. This study would support the notion that the regulator can also expand its mandate to ensure that the relationship with practitioners is not only limited to registration requirements and disciplinary issues, but also actively incorporate training, professional development, proactive guidance and parallel interaction. The recent first national conference and practitioner ‘roadshows’ hosted by the HPCSA are heartening developments in this regard (HPCSA 2019b). Actions such as these will hopefully lead to a closer relationship and collaborative efforts between the regulator and its registrants, and a greater willingness to engage in mutually beneficial processes.

Future research could be aimed at corroborating these findings with a larger sample of practitioners across a variety of disciplines. Additional research in the interdisciplinary fields of psychology, ethics and law could also improve our understanding of how to best protect the professions against false complaints, and how to reconsider the relationship between practitioners, the regulator, as well as the role of professional organisations and practitioner networks.

## Conclusion

There is no argument that not even the best professional standards and conscientious care can protect practitioners against a professional malpractice complaint. As the probability of complaints in modern-day practice is high, this report illuminates the need to instil effective and collaborative strategies between the regulator and professionals. Professions are self-regulating which implies that the professionals doing the regulating and those who are being regulated are affiliated. A revisioning of the relationship between the registration body and practitioners to include a more co-operative and educative engagement during a complaint process may eliminate the vulnerability, fear and inequality experienced by some practitioners. Processes could be examined to ensure scrupulousness, consistency, transparency and expediency. This will hopefully lead to more effective complaint management and achieving the often-elusive balance in the principles of justice and non-maleficence, simultaneously protecting the rights of the complainant and the well-being of the practitioner.

## Footnotes

1 Specific professional boards govern the various professions. The Board of Psychology manages professional misconduct complaints against psychologists.
